# Regional differentiation and extensive hybridization between mitochondrial clades of the Southern Ocean giant sea spider *Colossendeis megalonyx*

**DOI:** 10.1098/rsos.140424

**Published:** 2015-07-29

**Authors:** Lars Dietz, Claudia P. Arango, Jana S. Dömel, Kenneth M. Halanych, Avril M. Harder, Christoph Held, Andrew R. Mahon, Christoph Mayer, Roland R. Melzer, Greg W. Rouse, Andrea Weis, Nerida G. Wilson, Florian Leese

**Affiliations:** 1 Faculty of Biology and Biotechnology, Department of Animal Ecology, Evolution and Biodiversity, Ruhr University Bochum, Universitaetsstrasse 150, Bochum 44801, Germany; 2 Natural Environments Program, Queensland Museum, PO Box 3300, South Brisbane, Queensland 4101, Australia; 3 Auburn University, 101 Rouse Life Sciences Building, AL 36849, USA; 4 Department of Biology, Institute for Great Lakes Research, Central Michigan University, Mount Pleasant, MI, USA; 5 Alfred Wegener Institute, Helmholtz Center for Marine and Polar Biology, Am Alten Hafen 26, Bremerhaven 25768, Germany; 6 Zoological Research Museum Alexander Koenig, Adenauerallee 160, Bonn 53113, Germany; 7 Bavarian State Collection of Zoology—SNSB, Münchhausenstraße 21, Munich 81247, Germany; 8 Department Biology II, Ludwig-Maximilians-Universität München, Großhaderner Straße 2, Planegg-Martinsried 82152, Germany; 9 GeoBio-Center, Richard-Wagner-Straße 10, Munich 80333, Germany; 10 Scripps Institution of Oceanography, University of California, San Diego, 9500 Gilman Drive, La Jolla 92093-0202, CA, USA; 11 Western Australian Museum, Locked Bag 49, Welshpool DC, Western Australia 6986, Australia

**Keywords:** Antarctic, benthos, glacial refugia, phylogeography, Pycnogonida, speciation

## Abstract

Assessing the enormous diversity of Southern Ocean benthic species and their evolutionary histories is a central task in the era of global climate change. Based on mitochondrial markers, it was recently suggested that the circumpolar giant sea spider *Colossendeis megalonyx* comprises a complex of at least six cryptic species with mostly small and non-overlapping distribution ranges. Here, we expand the sampling to include over 500 mitochondrial COI sequences of specimens from around the Antarctic. Using multiple species delimitation approaches, the number of distinct mitochondrial OTUs increased from six to 15–20 with our larger dataset. In contrast to earlier studies, many of these clades show almost circumpolar distributions. Additionally, analysis of the nuclear internal transcribed spacer region for a subset of these specimens showed incongruence between nuclear and mitochondrial results. These mito-nuclear discordances suggest that several of the divergent mitochondrial lineages can hybridize and should not be interpreted as cryptic species. Our results suggest survival of *C. megalonyx* during Pleistocene glaciations in multiple refugia, some of them probably located on the Antarctic shelf, and emphasize the importance of multi-gene datasets to detect the presence of cryptic species, rather than their inference based on mitochondrial data alone.

## 1. Introduction

Species diversity in the marine Antarctic benthos is severely underestimated [1–3]. One of the main reasons for this problem is still the limited sampling of remote regions and habitats such as the continental slope [4]. Another major challenge is the presence of cryptic or overlooked species, i.e. species that are currently not distinguished morphologically but are genetically distinct (see [5] for a review). With the recent use of molecular techniques, in particular a fragment of the mitochondrial cytochrome c oxidase subunit I (COI) or ‘barcoding gene’, many highly divergent clades have been found and interpreted as different species (e.g. [6–10]). The discovery of new species with molecular tools has not only improved our knowledge about the true magnitude of biodiversity in the Antarctic, it has also challenged central biogeographic paradigms in the Southern Ocean: traditionally, it has been assumed that many Southern Ocean marine animal species have a broad circumpolar [11–13] and eurybathic [14] distribution. Identification of cryptic species with molecular-based tools in a variety of Antarctic invertebrates has questioned this concept as several of these cryptic species show a very strong regional differentiation, particularly in brooders with a holobenthic lifestyle (i.e. no planktonic dispersal stage, see [15] for a review). Lack of dispersal and isolation in independent glacial refugia during the Late Cenozoic ice-ages have been suggested as the main drivers of regional diversification and speciation [16–19]. However, some brooders with a regionally differentiated population structure were not found to contain cryptic species (e.g. the pycnogonid *Nymphon australe*[20,21]) while others with a planktonic dispersal stage have widely distributed cryptic species, such as the crinoid *Promachocrinus kerguelensis* [22]. In some species groups, several lineages occur in sympatry (e.g. [9,23]), suggesting that ecological speciation may play an important role. The role of bathymetry in speciation has been reported for other Southern Ocean invertebrates [24]. In some groups, morphological investigations support the distinction of previously unrecognized species that were identified with molecular data (e.g. [25–29]).

Most of these molecular studies, however, have been based only on mitochondrial genes. As several cases have been observed where mitochondrial and nuclear data disagree due to phenomena such as introgressive hybridization or sex-biased dispersal (reviewed in [30]), this can be misleading. Therefore, nuclear data should be studied as well before the existence of cryptic species can be established.

In this study, we analysed the diversity of the giant sea spider species *Colossendeis megalonyx* Hoek, 1881 using both nuclear and mitochondrial gene data. *C. megalonyx*is one of the most widespread pycnogonid species in the Southern Ocean [31], with a circumpolar distribution in Antarctic and Subantarctic waters and also found in South America, South Africa and Madagascar, from 3 to 4900 m depth [32]. Although other sea spiders are benthic brooders with paternal care, the reproductive mode of the entire Colossendeidae family is still unknown [33]. Because of its wide distribution and high morphological variability, it has often been questioned whether *C. megalonyx* is a single species [34], and several subspecies and putatively synonymous species have been described (e.g. [35,36]). However, no detailed systematic morphological study has been published yet.

A recent study by Krabbe *et al*. [37] investigated *C. megalonyx* from a molecular perspective. It was shown that COI sequences of *C. megalonyx* fall into six major clades with limited distribution ranges and with interclade genetic distances comparable to those of distinct species. However, the 96 samples included in that study covered only few areas (South Sandwich Islands, Elephant Island, Bouvet Island, Burdwood Bank). Here, we substantially expanded the dataset of Krabbe *et al*. [37] by adding COI data for over 300 specimens from the same areas as well as from other regions in South America, along the Scotia Arc, and from the West and East Antarctic shelf. We further included data from an additional locus, the nuclear ribosomal gene region internal transcribed spacer (ITS), for a subset of individuals. This region, which includes the gene for 5.8S rRNA as well as the non-coding ITS1 and ITS2, has been found to be useful to distinguish closely related species in many different animal groups (e.g. [38–40]), including pycnogonids [41]. With the new dataset, we tested (i) whether there are further overlooked mitochondrial clades additional to the six clades found by Krabbe *et al*. [37]; (ii) whether the proposed narrow distribution ranges of the clades were supported by the new data from many more regions; (iii) whether or not the nuclear data support the pattern revealed by the mitochondrial data; and (iv) whether *C. megalonyx* colonized the Antarctic from the Subantarctic or vice versa. We discuss the new findings in the context of marine Antarctic evolution during the Pleistocene glaciations.

## 2. Material and methods

**Figure 1.**
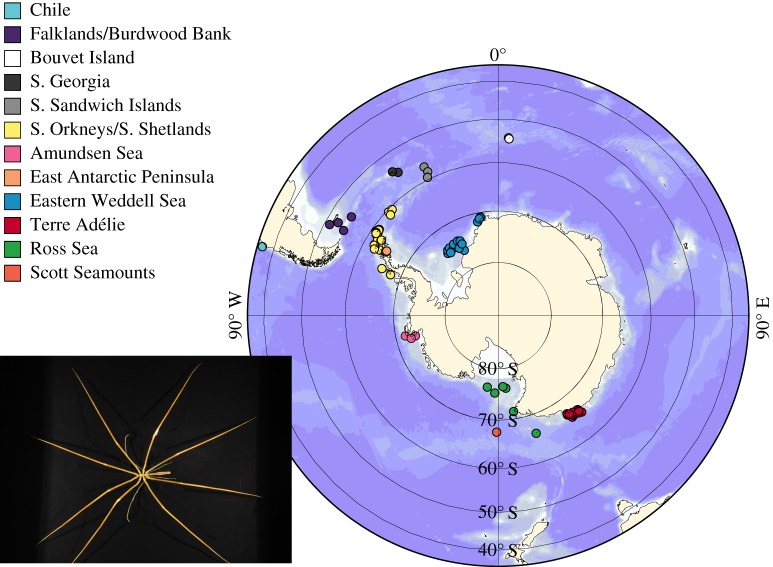
Map of the Southern Ocean with sampling sites of the specimens of *Colossendeis megalonyx* analysed in this study. Colours correspond to those in figures 3 and 4. For a detailed overview of samples and sampling sites, see electronic supplementary material, S1 and S2. Photo of *Colossendeis megalonyx*: Claudia P. Arango.

A 658 bp fragment of the mitochondrial COI gene was sequenced for a total of 418 putative *C. megalonyx* specimens from different parts of the Southern Ocean (see figure 1 for a map of the sampling sites) and for an additional 82 specimens belonging to other colossendeid species (table S1). Individuals were determined to species level with the keys of Child [34] and Pushkin [42] prior to completing any genetic analyses. DNA extractions were performed using the Qiagen DNeasy Blood & Tissue Kit following the manufacturer's protocol with the exception of using only 100 μl elution buffer (EB) to increase final DNA concentration. PCR for COI was performed as outlined by Krabbe *et al*. [33].

An approximate 1000 bp fragment of the ITS (18S–ITS1–5.8S–ITS2–28S) was sequenced for a subset of 76 *C. megalonyx* specimens and 34 other colossendeids. PCR was performed as follows: 94°C for 2 min, followed by 37 cycles of 94°C for 20 s, 55°C for 30 s and 65°C for 80 s, with a final extension at 65°C for 10 min. Primers used for PCR were ITSRA2 and ITS2.2 [43].

For both gene regions, the PCR mix consisted of 2 μl 10× HotMaster Taq Buffer (5Prime, Hilden, Germany), 2 μl of 2 mM dNTPs, 0.1 μl of 100 μM HCO or ITSRA2 primer, 0.1 μl of 100 μM LCO or ITS2.2 primer [43,44], 0.1 μl of 5 U μl^−1^ HotMaster Taq (5Prime, Hilden, Germany), 1 μl DNA (approx. 20 ng), filled up to 20 μl with sterile H_2_O. PCR products were purified with a 1 : 2 mix of Exo and FastAP for 15 min at 37°C followed by inactivation for 15 min at 85°C. Sequencing was performed at GATC Biotech (Cologne, Germany).

For COI, the colossendeid sequences from Krabbe *et al*. [37] (96 *C. megalonyx*, 19 from other species) and all sequences from GenBank that were identified as members of the Colossendeidae by BLASTn searches (37 *C. megalonyx*, 113 from other species) were added to the total resulting dataset for analyses.

For both gene regions, sequences were edited with Geneious v. 6.1.6 [45]. COI was aligned using MUSCLE [46] with the default parameters as implemented in Geneious, using eight iterations. The ITS region was aligned with MAFFT 7 [47] using the E-INS-I algorithm with a gap opening penalty of 1.53 and offset value 0. For COI, we verified that all codons could be translated into amino acids without stop codons using the invertebrate mitochondrial genetic code. For ITS, a version of the alignment with ambiguously aligned regions removed was produced with Gblocks 0.91b [48] using less stringent parameters (smaller blocks, gaps in final alignment allowed, less strict flanking positions). Bayesian phylogenetic analysis was performed with MrBayes v. 3.2.1 [49] using 5 000 000 MCMC generations, of which the first 25% were discarded as burn-in (test for convergence: split divergence less than 0.01). The most suitable model of molecular evolution for the analyses was selected with jModeltest v. 2.1.2 [50]. Maximum-likelihood analysis was performed with RAxML v. 7.03 [51] and support was assessed with 1000 rapid bootstrap replicates.

For COI, sequences were collapsed into haplotypes with the online Fabox haplotype collapse tool [52]. A p-distance matrix was created using MEGA v. 6.06 [53].

For species delimitation, a general mixed Yule coalescent (GMYC) analysis was performed. For this, a linearized tree of the haplotypes was calculated using BEAST v. 1.8 [54] with the model specified by jModeltest. Convergence and effective sampling size (ESS > 200) of parameter estimates were checked using Tracer v. 1.5 [55], and a consensus tree was calculated using TreeAnnotator v. 1.8 of the BEAST package and analysed with the SPLITS program available as a package for the statistical software environment R [56]. A Bayesian GMYC (bGMYC) analysis [57] with a threshold of 0.5 was also performed using the last 100 trees in the BEAST MCMC file. An additional test for presence of distinct clades was performed using the program ABGD [58] using Kimura-2-parameter (K2P) distances. Minimum spanning networks of haplotypes for the four largest clades (A, D1, E and I) were created with PopART (http://popart.otago.ac.nz, v. 1.7).

## 3. Results

After removal of poorly represented regions at the 3′ and 5′ end, the COI alignment had a length of 545 sites, of which 265 were variable and 226 parsimony-informative. The 549 *C. megalonyx* sequences grouped into 156 haplotypes. The ITS alignment had a total length of 1145 sites, of which 393 were variable and 293 parsimony-informative. The 76 ITS sequences of *C. megalonyx* grouped into 36 haplotypes. After removing ambiguously aligned regions from the ITS alignment with Gblocks, the number of bases was reduced to 965 sites, of which 313 were variable and 236 were parsimony-informative. The ITS alignment also contained several gaps. For COI and the cropped ITS alignment, the model *GTR*+I+G was chosen by jModeltest, while GTR + G was chosen for the full ITS alignment.

### 3.1 Species delimitation

#### 3.1.1 Cytochrome c oxidase subunit I data

**Figure 2.**
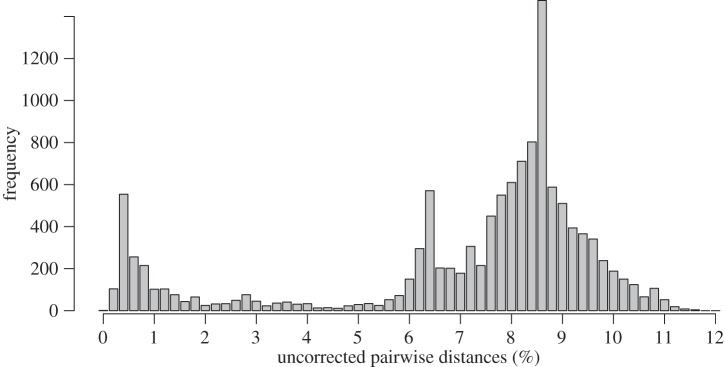
Histogram of uncorrected pairwise genetic distances for the COI fragment within *C. megalonyx*(all clades). Only unique haplotypes are used for calculating pairwise distances.

The COI data showed consistency with morphological identifications as specimens determined as *C. megalonyx* formed a clearly delimited monophyletic group. Only two specimens from Kerguelen initially determined as *C. megalonyx* grouped outside that clade, suggesting that they do not belong to the *C. megalonyx* complex. The K2P genetic distances showed a clear bimodal distribution, with a barcoding gap at approximately 4% (figure 2).

GMYC analysis showed that a single-threshold GMYC model was better than a single population model (*p*=5.5×10^−10^) and maximum-likelihood (ML) resulted in a number of 20 ML entities (confidence interval: 19–43), including the six clades already recognized by Krabbe *et al*. [37]. Material was available for 17 of the ML entities, while three clades (J, K, L) were only based on GenBank specimens. The number of samples in each clade ranged from one (clades J, M) to 161 (clade A). Average intraclade distances ranged from 0 to 1.9%, while interclade distances ranged from 2.7 to 12.5%. The same clades are also distinguished in the bGMYC analysis. These groupings are shown in table 1.

**Table 1.** Number of specimens per COI GMYC clade in each location. The total number may be larger than the sum of numbers for individual regions as some specimens lack locality information. Colours refer to those used in figure 1.

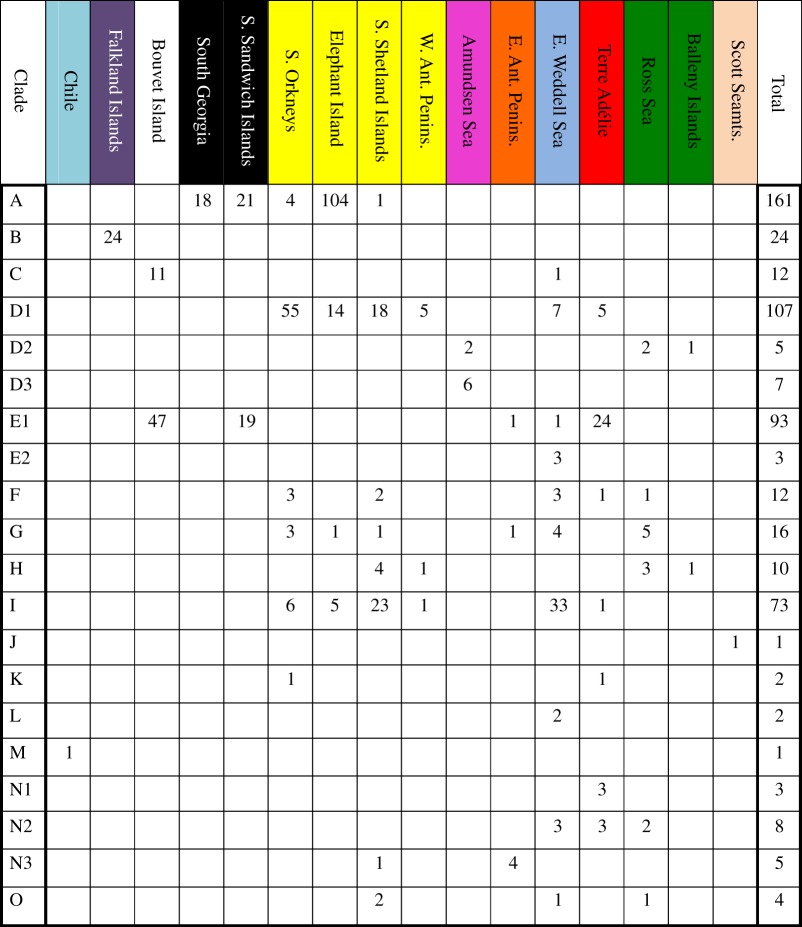

The ABGD analysis resulted in only 15 clades, here termed A–O. Clades D and N correspond, respectively, to three clades in the GMYC analysis, while clade E corresponds to two clades. Here these clades are named D1/D2/D3, E1/E2 and N1/N2/N3 (see table 2 for clade delimitations resulting from different approaches).

**Table 2.** Differences in COI clade delimitation in *C. megalonyx* based on single-threshold GMYC (same results as bGMYC) and ABGD. Numbers in rightmost column refer to ITS clades to which individuals from the respective COI clade are assigned. n/a refers to COI clades for which no ITS sequences were available.

clade	GMYC	ABGD	ITS group
A	A	A	IV, VI
B	B	B	I
C	C	C	I, II
D1	D1		II
D2	D2	D	n/a
D3	D3		n/a
E1	E1		II, III
E2	E2		II
F	F	F	VI
G	G	G	III, V
H	H	H	IV
I	I	I	IV, V
J	J	J	n/a
K	K	K	n/a
L	L	L	n/a
M	M	M	n/a
N1	N1		n/a
N2	N2	N	III
N3	N3		III
O	O	O	n/a

**Figure 3.**
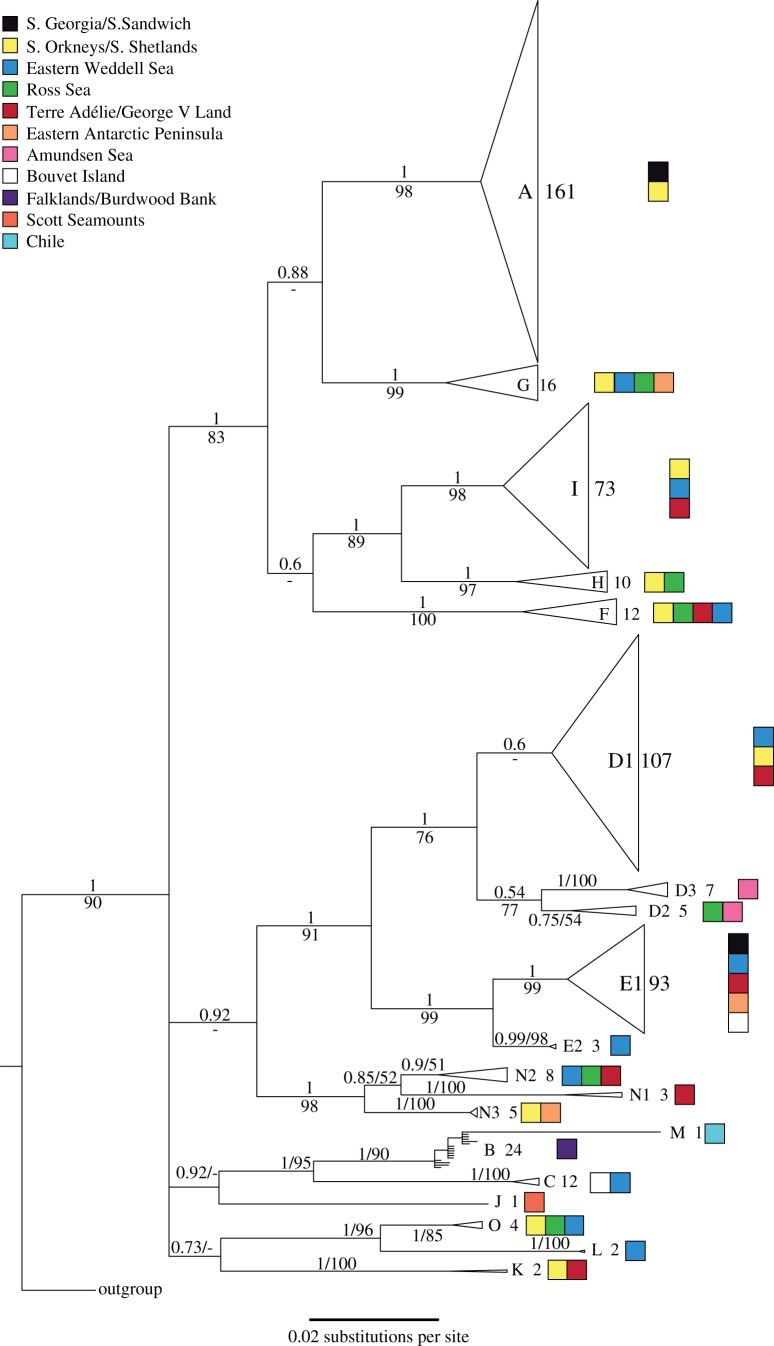
Bayesian phylogenetic tree of *C. megalonyx* COI sequences. Clades recognized by GMYC/bGMYC analysis have been collapsed. Numbers above branches are posterior probabilities, numbers below branches are bootstrap percentages for the maximum-likelihood analysis. Numbers beside clade names show number of samples. Colours indicate geographical origin of samples.

Bayesian phylogenetic analysis recovered most of the single-threshold GMYC groupings as monophyletic, although B was paraphyletic with respect to M (figure 3). While many interclade relationships are poorly resolved, several clades formed strongly supported monophyletic groups, namely A+F+G+(H+I),(B+M)+C,D+E and L+O. Interestingly, the clades B (Falkland Islands) and M (Chile) formed a South American group clustering inside the mostly Antarctic *C. megalonyx* complex as sister to clade C (Bouvet, Eastern Weddell Sea). Similar results were found in the maximum-likelihood analysis, except that clade D1 was found to be paraphyletic with respect to D2 and D3.

#### 3.1.2 Internal transcribed spacer data

**Figure 4.**
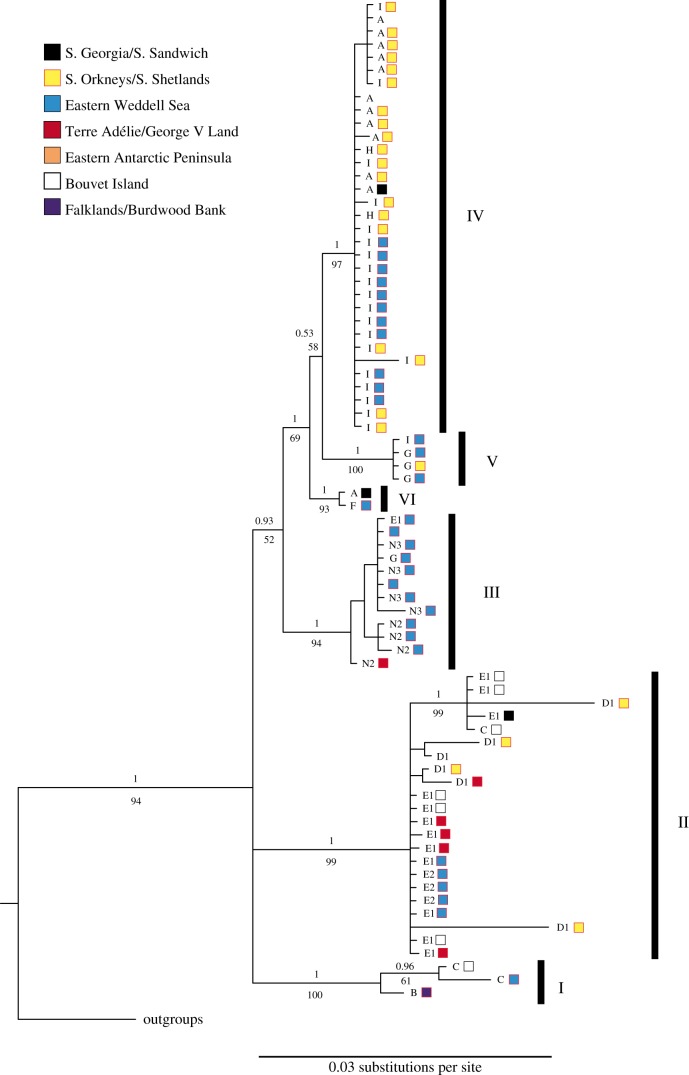
Bayesian phylogenetic tree of *C. megalonyx* ITS sequences. Numbers above branches are posterior probabilities, numbers below branches are bootstrap percentages for the ML analysis. Letters on branches refer to COI clade assignment of the respective specimen. Colours indicate geographical origin of samples. Numbers I to VI refer to the six ITS groups identified.

The ITS phylogenetic tree showed a differentiation into six distinct monophyletic groups named I–VI (figure 4), which mostly correspond to larger groupings of different COI clades. The analysis of the dataset cropped with Gblocks resulted in a slightly different phylogenetic tree, but the differentiation into six groups did not change. There was no differentiation between specimens belonging to different COI clades within these groups. As an example, group IV included individuals belonging to COI clades A, H and I, but those clades could not be distinguished by ITS sequences. Group II showed strong intra-group variation, but no division into the COI clades D1, E1 and E2 was found. In some cases, there were also discrepancies in assignment to larger groups between COI and ITS. Group II mostly included individuals from clades D and E, but also one clade C individual. Group III included individuals from clades N2 and N3 as well as one each from clades E and G. Individuals from COI clade I were found in both groups IV and V.

### 3.2 Phylogeographic analysis

#### 3.2.1 Cytochrome c oxidase subunit I data

Most of the clades are geographically widely distributed, and eight of them are found in both East and West Antarctica (table 1). Of the clades represented by more than three specimens, only clade A (*n* = 161) is restricted to the Scotia Arc, while clade B (*n*=24) is restricted to the Falklands/Burdwood Bank. Even representatives of very rare clades, such as K (*n*=2) and O (*n*=4), are found in widely distant regions. None of the clades found in this study seem to show a truly circumpolar distribution as they are notably absent in entire well-sampled regions in our sampling. For example, clade D1, which is known from the South Orkneys/South Shetlands, the Eastern Weddell Sea and Terre Adélie/George V Land, was not found in South Georgia or the South Sandwich Islands. Specimens of distinct clades often occur sympatrically in the same regions, especially around the South Orkneys (six clades) and the South Shetlands (seven clades), and even occur at the same sampling station as in the case of station 11740 in the South Shetlands where 22 individuals from five different clades were collected.

**Figure 5.**
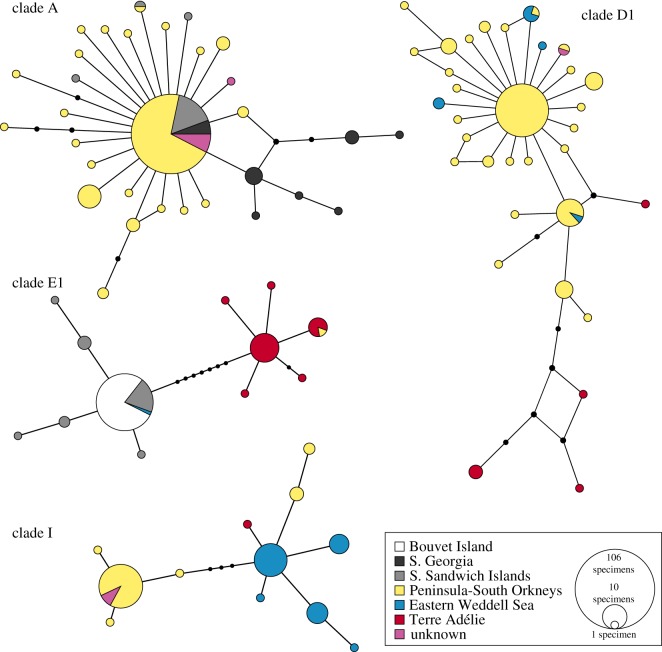
Haplotype network for *C. megalonyx* clades A, D1, E1 and I based on the mitochondrial COI gene. Sizes of circles are proportional to number of individuals per haplotype. Colours indicate geographical origin of samples. Black dots represent hypothetical haplotypes.

The haplotype network for clade A (figure 5) shows a ‘star-like’ pattern centred around the common haplotype A-2 (*n*=106). All other 27 haplotypes from the South Sandwich, South Orkney and South Shetland Islands differ from it by only one to three substitutions and are known from only one to three samples (except for A-7 from Elephant Island with *n*=7). Compared to all other regions, there is much more variability in South Georgia, with haplotype A-2 occurring less frequently compared with other regions (figure 5).

For clade D1, there is a clear differentiation between eastern (Terre Adélie/George V Land, *n*=6) and western (South Orkneys/South Shetlands/Western Antarctic Peninsula/Eastern Weddell Sea, *n*=101) specimens, with the latter forming a star-like pattern that is mostly due to the large number of specimens with haplotype D1–2 from the South Orkneys. However, a single specimen from East Antarctica (haplotype D1–14) groups closer to the western specimens (figure 5).

In clade E1, there is also a strong differentiation between western (South Sandwich/Bouvet/Eastern Weddell Sea, *n*=67) and eastern (Terre Adélie/George V Land, *n*=24) samples, with eight steps in between. Interestingly, all specimens from Bouvet (*n*=43) belong to a single haplotype (E1-1), which is also the most common in the South Sandwich Islands. One specimen from the Eastern Antarctic Peninsula has a haplotype that otherwise occurs in Terre Adélie specimens (figure 5).

COI clade I is divided into two clusters, with four steps in between. One cluster is found only in the South Orkneys/South Shetlands, the other one occurs in the South Shetlands (incl. Elephant Island) as well as in the Eastern Weddell Sea and in Terre Adélie. However, no haplotypes are shared between the Eastern Weddell Sea and Scotia Arc locations. Representatives of both clusters were found in the same sampling stations (11719 and 11740).

#### 3.2.2 Internal transcribed spacer data

Notably, the ITS sequences of individuals from the same locality often group together even if they belong to different COI clades. As an example, sequences of specimens from Station 260 on the Eastern Antarctic Peninsula, which belong to the mitochondrial clades E1, G and N3, form a cluster in the ITS phylogenetic tree. One clade C individual from Bouvet has an ITS sequence grouping with those of individuals in clade E1 from the same location.

## 4. Discussion

### 4.1 Number of mitochondrial groupings

Based on the COI data, it appears that the *C. megalonyx* complex consists of about 15–20 distinct unrecognized species. Different methods (GMYC and ABGD) disagree on the exact delimitation of some clades. These mostly involve those cases in which the distances are at an intermediate level falling into the barcode gap (about 2–5%), i.e. the clades D1+D2+D3,E1+E2 and N1+N2+N3. Interpretation of these as one, two or three distinct groupings should be considered ambiguous. In all other cases, the two methods agree, therefore showing a clear distinction between inter- and intraclade divergence levels, i.e. the presence of a barcoding gap.

### 4.2 Geographical distribution of mitochondrial groups

Specimens of some of the different clades recovered show a narrow distribution range, others are widely distributed and occur in sympatry. This stands in contrast to the findings of Krabbe *et al*. [37], who analysed only 96 specimens with more limited geographical sampling than in this study. The results here agree with several other studies on Antarctic benthic invertebrates (e.g. [9,20,22]) proposing circumpolar distributions of species based on molecular data. It should be noted that at least two clades exhibit obvious morphological differences from all others, namely clade C, which lacks pigmented eyes, and clade F, which includes animals significantly larger than all others examined. Lack of eyes has been previously reported for the (sub)species *C*. (*megalonyx*) *orcadense* [36,59] known from the South Orkneys, South Africa and Madagascar, while clade C is restricted to Bouvet Island and the Weddell Sea slope in our samples. If several species coexist sympatrically, it is to be expected that they exhibit ecological differences. Therefore, we expect that, if the mitochondrial clades are indeed distinct species, a detailed study would reveal noticeable morphological or physiological [23] differences.

### 4.3 Implications of the nuclear data

As shown in the results, there are several instances where the ITS data are incongruent with the COI data. This could be explained by retention of ancestral polymorphisms or by intragenomic polymorphism in ITS, as has been described for other arthropods [60,61]. However, the differences observed between different ITS groups in our study are generally higher than observed in those cases. Besides, we did not detect large amounts of conflicting signals in our sequence electropherograms, which would be expected in the case of polymorphic ITS sequences. In addition, our analysis of assembled 454 sequence data of the ITS gene region obtained from a preceding project [62] with a coverage of 18.6× showed no evidence for multiple intragenomic variants. The clustering of ITS sequences from individuals found geographically close to each other would also be difficult to explain by the presence of polymorphisms. Therefore, we propose that the best explanation for our results is hybridization between different COI clades. As there seems to be extensive hybridization between related COI clades such as A, H and I, they cannot be considered distinct species. Between the larger monophyletic groupings such as D+E or A+F+G+H+I, hybridization events appear to be rare, and they may be recognized as distinct species. The analysis of the nuclear gene H3 for specimens belonging to clades A–F included in Krabbe *et al*. [37] further supports the validity of the larger groups A+F, B+C and D+E [63], but, shows no differentiation of the COI clades within those groups.

Occurrence of very similar ITS sequences in specimens from the same site belonging to different COI clades indicates that hybridization is still ongoing, i.e. animals with very divergent mitochondrial genomes seem to belong to the same gene pool. Therefore, ITS data provide evidence that the number of 15–20 cryptic species inferred from the COI sequences could be an overestimation. While the distinct COI clades probably differentiated in isolation from each other, possibly as a consequence of temporary isolation during earlier glaciation periods, apparently there have been no barriers to hybridization after these clades came into contact again. Hybridizing clades are up to 8% divergent based on COI, which according to standard molecular clocks for arthropod taxa [64,65] would imply a divergence time of more than a million years ago. If that was the case, a long period of independent evolution of the COI clades did not lead to reproductive isolation. Despite the incongruences between ITS and COI clades, larger monophyletic groups recognized with COI mostly agree with those recognized with the nuclear gene regions H3 and ITS. With some exceptions, those groupings seem to be largely reproductively isolated and therefore could be regarded as distinct species. The number of known species within the *C. megalonyx* complex would then be possibly about five to seven. Limited hybridization between them resulting in mitochondrial–nuclear discordance is similar to that reported for other groups of related species [30].

The ITS data also provide information on the population history in some regions. For instance, within ITS group II, only three out of six examined clade E1 individuals from Bouvet Island show ITS sequences highly similar to those of specimen PB_E002 from the South Sandwich Islands, while the others group more basally within group II. All clade E1 specimens from Bouvet share a single COI haplotype that is also found in South Sandwich and the Eastern Weddell Sea. This might indicate that not all of the Bouvet population originated from a single colonization event, as would be inferred from the COI analysis. Instead, there may have been several different colonizations of Bouvet, and the mitochondrial haplotype originating in a recent dispersal from South Sandwich or the Eastern Weddell Sea seems to be fixed in the population while the ITS region retains more variability. In general, fixation of mitochondrial gene variants is expected to occur faster than in nuclear DNA due to the smaller effective population size [66].

On the one hand, our results contrast with those found in some other marine benthic organisms, including pycnogonids [41], nudibranchs [23], Antarctic isopods [67] and amphipods [9] in which mitochondrial and nuclear data agree on the delimitation of unrecognized species. On the other hand, Hemery *et al*. [22] found results similar to ours in the Antarctic crinoid *Promachocrinus kerguelensis*, in which mitochondrial markers and ITS defined two major groups but further differentiation into seven mitochondrial clades was not supported by ITS data. However, in *P. kerguelensis* the COI divergence among clades was lower than in the *C. megalonyx* complex, and the lack of resolution with ITS may be due to a taxon-specific lower mutation rate in *P. kerguelensis*. Similar results also occur in species with significantly different life histories, such as the stonefly *Dinocras cephalotes* [68], in which two highly divergent COI lineages occur in sympatry but no differentiation was found with nuclear data. In many cases, coexistence of highly divergent mitochondrial lineages within a single species can be explained by introgressive hybridization with other species (e.g. [69]). However, in this study, all mitochondrial haplotypes found within the *C. megalonyx* complex clearly form a monophyletic group and no introgression from other colossendeid species was found.

Environmental change may lead to the breakdown of ecological barriers between reproductively isolated groups and therefore to ‘speciation reversal’ [70]. Although this has been demonstrated mostly for anthropogenic change, glaciations may possibly have similar effects on Antarctic shelf fauna by restricting distributions of benthic organisms to small refugia. This would imply that previously isolated lineages collapsed into a hybrid swarm, which may have led to strong mitochondrial–nuclear discordance. The question arises why such a pattern is not present in other Antarctic species that have been investigated. Possibly, due to differences in environmental conditions between glacial refugia, selection would have led to different adaptations [23]. While in some cases these differences were sufficient for reproductive isolation, this was apparently not the case for the *C. megalonyx* radiation.

### 4.4 Out of Antarctica hypothesis

We found that there is a monophyletic ‘Subantarctic’ grouping restricted to South America, nested within the Antarctic *C. megalonyx* complex. This pattern suggests that the Subantarctic was colonized from the Antarctic and not vice versa, as also found e.g. in cephalopods [71]. As the holotype of *C. megalonyx* is a specimen from the South American shelf [72], it can be expected to belong to the Subantarctic group, to which the species name should therefore be restricted. *C. megalonyx* would then lose its status as an Antarctic pycnogonid, as the species would be restricted to the Subantarctic and possibly to South America.

### 4.5 Multiple *in situ* glacial refugia

In addition to biogeographic and systematic questions, this study also provides important data to the debate on Antarctic glacial refugia [19], in particular on their putative localities. Our results provide no support for the hypothesis that *C. megalonyx* *sensu lato* survived the glaciations *ex situ* in refugia in the Subantarctic shelf regions, as no sequences from Antarctic specimens nest within the Subantarctic clades. However, we lack samples from several non-Antarctic areas where *C. megalonyx* has been found, such as South Africa, Kerguelen and the New Zealand Subantarctic islands. There is good evidence that South Georgia acted as a refugium for clade A, as shown by the much greater haplotype diversity arguing against a recent expansion, in contrast to the more southern Scotia Arc islands. A similar pattern was recently found by González-Wevar *et al*. [73] for the limpet *Nacella concinna*. As the geological evidence suggests that South Georgia was not fully glaciated during the Last Glacial Maximum (LGM) [74], the South Georgia shelf could plausibly have been a refugium for shelf-inhabiting taxa, which is in good agreement with the results of a pioneering species distribution modelling study on Southern Ocean shrimps [75].

The hypothesis that the shelf was recolonized from the deep sea after the LGM cannot be rejected by our data, as we have only few samples from deeper than 1000 m. However, we consider it unlikely, as circumpolar survival in the deep sea would lead to greater genetic homogeneity across regions and lack of signatures for recent expansion. Such a pattern is found in the shrimp *Nematocarcinus lanceopes* [76], but not in our data for *C. megalonyx*.

The hypothesis most consistent with our data is the *in situ* survival in ice-free refugia, which were probably located at polynyas (temporary ice-free ocean regions) as suggested by Thatje *et al*. [19]. Because of the strong intraclade regional differentiation in *C. megalonyx*, seen e.g. within clades D1 and E1, it seems likely that these clades survived in more than one refugium during the LGM, spreading from there and in some cases (clade I) coming into secondary contact. Molecular evidence for *in situ* survival on the Antarctic shelf has recently been reported for the broadly distributed sea spider *Austropallene cornigera* [77] and other invertebrates [78]. Our data support dispersal via the Antarctic Circumpolar Current (ACC) at least in the case of clade E1, which may have colonized Bouvet from the South Sandwich Islands, indicating a relatively recent (only one haplotype known from Bouvet) eastward dispersal in latitudes dominated by the ACC. However, the same haplotype also occur in the deep Weddell Sea, which suggests that Bouvet could also have been colonized from the south via the deep sea. Survival in multiple refugia would indicate that interclade splits precede the LGM, and probably occurred during earlier Pleistocene glaciations or even earlier.

In a few cases, we observe the same haplotype in geographically widely separated regions, such as a clade E1 haplotype (E1–3) that occurs both in the Antarctic Peninsula and Terre Adélie. This has also been observed in other invertebrates without a planktonic stage [9,20,79] and might be explained by rafting on floating material carried by currents, including ice. Pycnogonids have also been observed swimming [80].

The strong regional differentiation, which apparently persisted since the LGM, is typical of benthic brooding organisms with limited dispersal capability. Adult pycnogonids are almost exclusively benthic, the reproduction mode of colossendeids is unknown and no larvae have been recorded from plankton samples. The distribution of *C. megalonyx* contrasts with that of benthic invertebrates with planktonic larvae such as the crinoids *Promachocrinus kerguelensis*, whose lineages mostly show a truly circumpolar and sympatric distribution [19].

## 5. Conclusion

Our largely expanded dataset supports the hypothesis that *Colossendeis megalonyx* is a complex of several overlooked species that radiated during the Pleistocene in multiple refugia in the Antarctic. Many of the species within the *C. megalonyx* complex show broad geographical distribution ranges. However, analysis of highly variable nuclear data in addition to mitochondrial COI gene data suggests that the number of actual overlooked species is smaller than the number of mitochondrial clades. These findings highlight the importance of including independent nuclear markers in species delimitation analyses. The taxonomy of the *C. megalonyx* complex may be further clarified by including nuclear data from other genes as well as morphological data. Next-generation sequencing technologies, which have the potential to sequence large numbers of loci at once, could be particularly useful in resolving this and similar questions.

## Supplementary Material

ESM1: Sampling sites details

## Supplementary Material

ESM2: GenBank sequences included in this study

## Data accessibility

All new sequence data have been deposited in GenBank (accession nos. KT201695–KT202231 and KT215646–KT215755). Alignments and tree data have been submitted to Treebase: http://purl.org/phylo/treebase/phylows/study/TB2:S17858
